# Thoracoscopic and Transmanubrial Approach for Mediastinal Tumor Extending to the Neck

**DOI:** 10.1016/j.atssr.2023.05.003

**Published:** 2023-06-02

**Authors:** Shohei Mori, Makoto Odaka, Maki Oh, Rintaro Shigemori, Naoki Toya, Takashi Ohtsuka

**Affiliations:** 1 Department of Surgery, The Jikei University Kashiwa Hospital, Chiba, Japan; 2 Division of Thoracic Surgery, Department of Surgery, The Jikei University School of Medicine, Minatoku, Tokyo, Japan

## Abstract

The combination of thoracoscopic and transmanubrial approaches provides good visualization and maneuverability of the cervicothoracic junction area. Here we report the case of a young woman with a mediastinal tumor extending to the neck who first underwent a thoracoscopic approach followed by a transmanubrial approach. This procedure was superior in terms of technical advantages and preserved function as well as minimally invasive and cosmetically pleasing. The preceding thoracoscopic approach made it easier to understand the location relationships between the tumor and anatomic structures in the transmanubrial approach. This combined procedure is appropriate for mediastinal tumors extending to the neck.

There are various surgical approaches for tumors at the cervicothoracic junction area, and the approach depends on several factors, such as tumor localization, invasion into the surrounding organs, and possibility of concomitant resection.^1^ The transmanubrial approach (TMA) provides good visualization of the neck and thoracic outlet and preserves sternoclavicular joint stability.^2^ However, a separate approach to the thoracic cavity is necessary if the mediastinal tumor extends far into the thoracic cavity. As a less invasive approach to the thoracic cavity, a combination of thoracoscopic approach and TMA for mediastinal tumors in the apex of the thoracic cavity and superior sulcus tumor has been reported.^3^^,^^4^

A 16-year-old Asian girl presented with an abnormal chest shadow on a chest radiograph during a health examination. She had no relevant symptoms or medical history. Chest computed tomography and magnetic resonance imaging revealed a left superior mediastinal lipoid tumor (maximum diameter, 12 cm) extending to the neck and circumferentially surrounding the internal jugular vein (Figure 1). The tumor was a suspected liposarcoma or lipoma, for which the patient underwent complete surgical resection.

**Figure 1:**
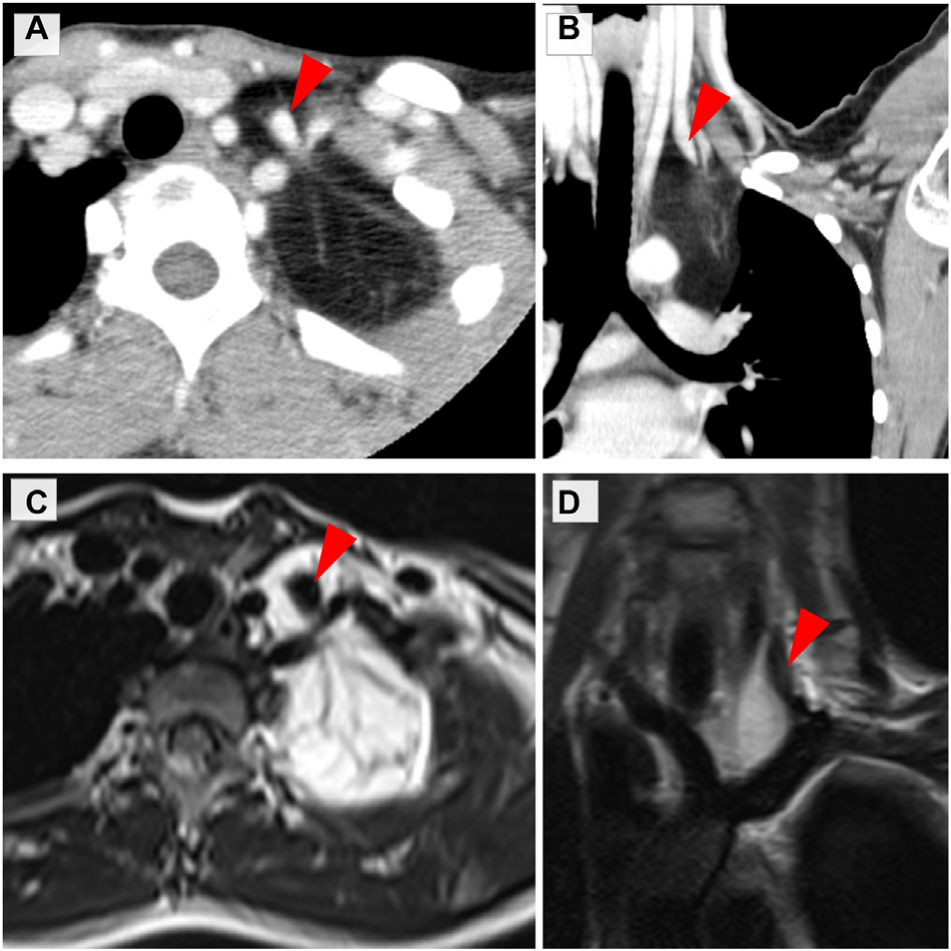
(A, B) Chest computed tomography and (C, D) magnetic resonance imaging revealed a left superior mediastinal lipoid tumor extending to the neck and circumferentially surrounding the internal jugular vein (arrowhead).

First, the thoracoscopic approach was preceded by the lateral position for good visualization and maneuverability of the superior mediastinum and the apex of the thoracic cavity as well as the hilum of the lung (Video). The access ports were the second and fourth intercostal spaces on the anterior axillary line, the fourth intercostal space on the midaxillary line, and the seventh intercostal space on the posterior axillary line. The tumor protruded widely from the superior mediastinum; however, the vertebral bodies and sympathetic trunks were intact. Because the location of the vagus and phrenic nerves around the tumor was unclear, the nerves were first identified at the pulmonary hilum, dissected from the tumor, and traced to the thoracic outlet (Figure 2A). The tumor was dissected from the aortic arch, common carotid artery, subclavian artery, and dorsal left brachiocephalic vein, and the portion contiguous to the neck was followed (Figure 2B). The wounds of the access ports were closed, and a chest tube was placed in the thoracic cavity.

**Figure 2:**
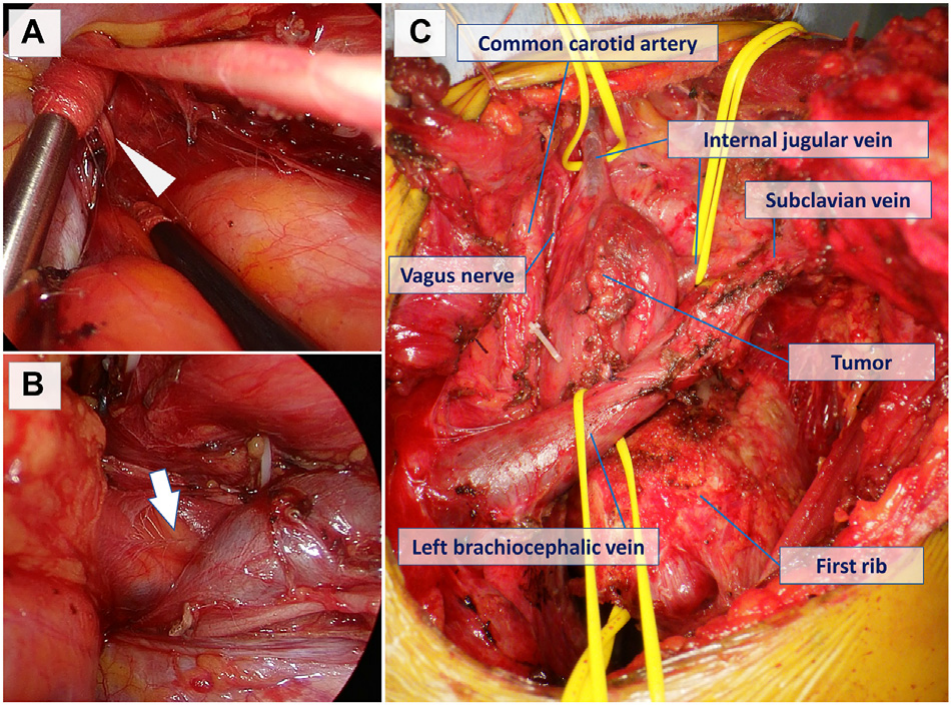
(A) The vagus nerve (arrowhead) was dissected from the tumor and traced to the thoracic outlet. (B) The tumor was dissected from the aortic arch, common carotid artery, subclavian artery, and dorsal left brachiocephalic vein, and the portion contiguous to the neck (arrow) was followed. (C) The transmanubrial approach was performed with the patient in the supine position, and the left brachiocephalic vein, left internal jugular vein, left subclavian vein, vagus nerve, and phrenic nerve were then exposed in the neck.

Second, TMA was performed with the patient in the supine position; the left brachiocephalic vein, left internal jugular vein, left subclavian vein, vagus nerve, and phrenic nerve were exposed in the neck (Figure 2C), and their continuity with the anatomic structures exposed in the thoracic cavity was confirmed. As the left internal jugular vein was invaded by the tumor, it was resected in a combined manner, and the tumor was removed through the TMA wound. The total operative time was 430 minutes (172 minutes for the thoracoscopic procedure, 206 minutes for the TMA procedure), and the blood loss was 20 mL.

The patient was discharged on postoperative day 11 without complications, such as phrenic nerve paralysis, recurrent nerve paralysis, or Horner sign. Wound healing was excellent at 1 month postoperatively (Figures 3A, 3B). On pathologic examination, the tumor was diagnosed as a lipoma without malignant findings (Figure 3C), and the internal jugular vein was surrounded by but not invaded by tumor cells (Figure 3D).

**Figure 3:**
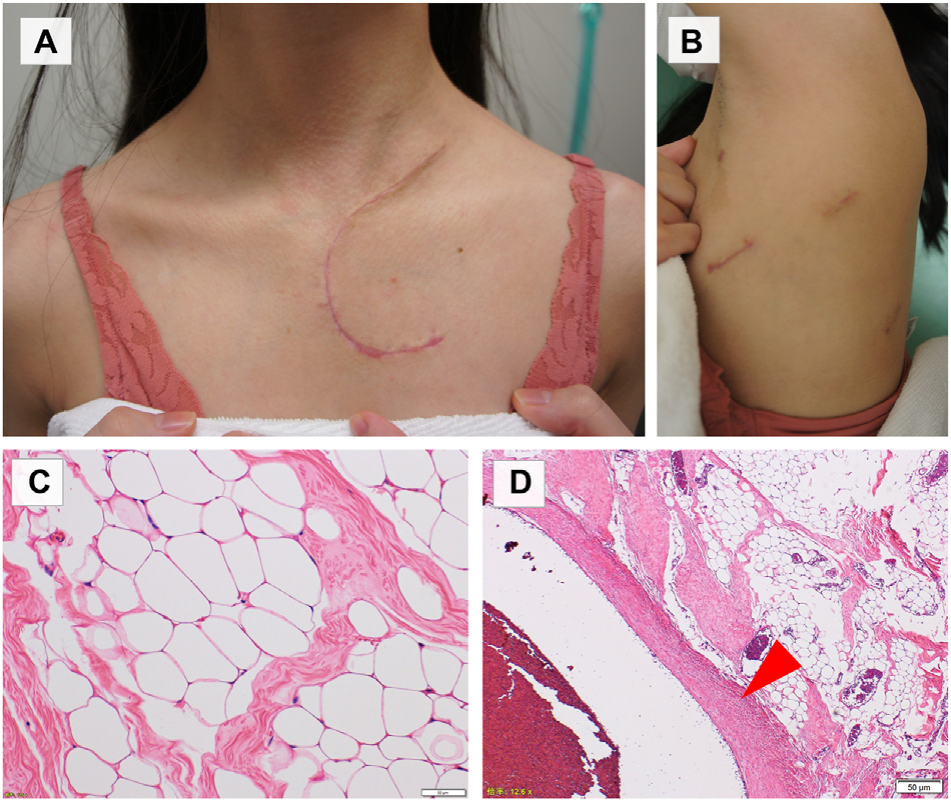
(A, B) Wound healing was excellent at 1 month postoperatively. (C) On pathologic examination, the tumor was diagnosed as a lipoma without malignant findings. (D) The internal jugular vein (arrowhead) was surrounded by but not invaded by tumor cells.

## Comment

This case provides two important clinical issues regarding the surgical approach for mediastinal tumors extending to the neck. First, the thoracoscopic approach has technical advantages. Second, the preceding thoracoscopic approach facilitates our understanding of the location relationships between the tumor and anatomic structures during TMA.

The thoracoscopic approach has several technical advantages. In this case, the thoracoscopic approach provided good visualization and maneuverability of the superior mediastinum and apex of the thoracic cavity. Although TMA is suitable for tumors at the cervicothoracic junction and preserves the stability of the sternoclavicular joint, a thoracic approach is necessary when the tumor widely extends into the thoracic cavity. Some successful cases have been reported using a combination of the thoracoscopic approach and TMA for mediastinal tumors at the cervicothoracic junction area and superior sulcus tumors.^3^^,^^4^ This case, different from the previous reports, was a huge mediastinal tumor extending from the pulmonary hilum to the neck, and the location of the vagus and phrenic nerves around the tumor was unclear. The characteristic point of the thoracoscopic approach was also that both nerves were first identified at the pulmonary hilum, dissected from the tumor, and traced to the thoracic outlet. This maneuver successfully avoided functional damage to the nerves. In addition to its technical advantages, the thoracoscopic approach is superior in terms of minimally invasive and cosmetic features. A combination of the thoracoscopic approach and TMA is appropriate for mediastinal tumors extending to the neck.

In addition, the preceding thoracoscopic approach facilitated our understanding of the location relationships between the tumor and anatomic structures during TMA. Considering the order of the thoracoscopic approach and TMA in this combined procedure, the thoracoscopic approach was used to assess the tumor’s resectability; to expose the tumor, vessels, and nerves in the thoracic cavity whenever possible to facilitate the continuity and location relationships of the tumor and anatomic structures in the neck; and to remove the huge tumor through the TMA wound. Although a change in position was necessary, the lateral position was chosen for the thoracoscopic approach; in other words, the reason for not choosing two approaches with the same hemilateral position and in the same disinfected field was that the thoracoscopic approach with the lateral position also allowed easy access to the hilum of the lung and posterior area of the superior mediastinum around the vertebral bodies and sympathetic trunks. The operative findings showed that this area was intact; however, the preoperative evaluation was not definitive.
